# Impact of lack-of-benefit stopping rules on treatment effect estimates of two-arm multi-stage (TAMS) trials with time to event outcome

**DOI:** 10.1186/1745-6215-14-S1-O2

**Published:** 2013-11-29

**Authors:** Babak Choodari-Oskooei, Max KB Parmar, Patrick Royston, Jack Bowden

**Affiliations:** 1 MRC Clinical Trials Unit, London, UK; 2 MRC Biostatistics Unit, Cambridge, UK

## Background

In 2011, Royston et al. described technical details of a two-arm, multi-stage (TAMS) design. The design enables a trial to be stopped part-way through recruitment if the accumulating data suggests a lack of benefit of the experimental arm. Such interim decisions can be made using data on an available `intermediate' outcome. At the conclusion of the trial, the definitive outcome is analysed. Typical intermediate and definitive outcomes in cancer might be progression-free and overall survival, respectively. In TAMS designs, the stopping rule applied at the interim stage(s) affects the sampling distribution of the treatment effect estimator, potentially inducing bias that needs addressing.

## Methods

Using simulations, we quantified the bias in the treatment effect estimator in TAMS trials for different designs. We also retrospectively `re-designed' completed cancer trials as TAMS trials and used the bootstrap to quantify bias.

## Results

In trials in which the experimental treatment is better than the control and which continue to their planned end, the bias in the estimate of treatment effect is small and of no practical importance. In trials stopped for lack of benefit at an interim stage, the treatment effect estimate is biased at the time of interim assessment. This bias is markedly reduced by further patient follow-up and reanalysis at the planned `end' of the trial.

## Conclusions

Provided that all patients in a TAMS trial are followed up to the planned end of the trial, the bias in the estimated treatment effect is of no practical importance. Bias correction is then unnecessary.

